# In Chains

**Published:** 1880-09

**Authors:** 


### "IN CHAINS."

Who is more in bondage than habitual tobacco users? either the chewer or smoker?

It would hardly do to say that the user of tobacco has lost all sense of, or appreciation, for the sensibility or feelings of other people, and yet in almost all cases their acts would seem to indicate this; yet it is more charitable to assume that their sense in this direction is wholly dominated by the filthy habit, and completely led captive, not always willingly, however, for many do bemoan their bondage. "In Chains" does not convey the full force of the habit, for bind a man with chains of iron and fasten with hooks of steel and he will at once set about freeing himself, and usually will accomplish it if let alone. Not so with the tobacco habit; the victim cannot free himself nor can his friends do it for him. There is no file or cold-chisel sharp enough and hard enough to cut the links of that chain.

The remark is sometimes made that the "tobacco user is filthy as a dog." Well, that is a slander upon the dog; and to make it at all applicable, just imagine a good sized dog with a quid of tobacco about the size of a walnut, chewing it vigorously till his free flow of saliva was thoroughly impregnated with the tobacco essence, and then ejecting it in streams in all directions, quite regardless of surroundings. Then the comparison may do. But who ever heard of a dog doing that? There is but one other animal, so far as I now remember, that can make itself so absolutely offensive as the thoroughly saturated tobacco user, and that is a ——————

If the tobacco habit had been in existence in the days of Moses the victim would certainly have been set out with the unclean animals.

Now these words are not for the irrevocably inthralled, but for those who are, or may be, beginners, may for some cause or other take up the practice.

Perhaps few, if any, begin the use of tobacco with the expectation or thought that they will become absolute slaves to it, and yet this is the rule with those who for any considerable time continue its use. If the end could be seen from the beginning there would be few, if any, who would enter upon such a course.

Of all men, the physician and dentist should most certainly abstain from the use of tobacco and for various reasons. First, that they should be as acceptable to those with whom they come in contact as possible. Second, that they may have a clear brain and sharp perception and good judgment. And third, that the nervous system may be under the most perfect control, and so manual execution be most perfect.

All persons are not alike susceptible to the disabilities that usually attach to this habit. While a few seem to go along for a long time with apparent impunity, the great majority suffer severely, and thousands are brought to an untimely end, after a life more or less inefficient. Then, boys, those of you who have begun *stop it*, if you can; and if you are yet free, then by every consideration remain free so far as tobacco is concerned.

